# Relationship between sex hormone levels, bone mineral density and bone turnover markers in healthy moroccan men: a cross-sectional study

**DOI:** 10.11604/pamj.2015.22.206.6066

**Published:** 2015-11-02

**Authors:** Aissam El Maataoui, Asmae Benghabrite, Abdellah El Maghraoui, Layachi Chabraoui, Zhor Ouzzif

**Affiliations:** 1 Mohamed V Souissi University, Faculty of Medicine and Pharmacy, Biochemistry Department at the Military Hospital, Rabat, Morocco; 2 Morocco Ministry of Health, Rabat, Morocco; 3 Mohamed V Souissi University, Faculty of Medicine and Pharmacy, Rheumatology Department at the Military Hospital, Rabat, Morocco; 4 Mohamed V Souissi University, Faculty of Medicine and Pharmacy, Biochemistry Department at the Ibn Sina Hospital, Rabat, Morocco

**Keywords:** Male osteoporosis, sex hormones, vitamin D, Bone remodeling markers

## Abstract

**Introduction:**

Gonadal steroid hormones play a crucial role during skeletal growth and maturation in both men and women. The aim of this study is to evaluate the relationship of sex hormone levels, bone mineral density and biochemical markers of bone turnover in healthy Moroccan men.

**Methods:**

142 Moroccan men who had no previous diagnosis of osteoporosis were enrolled prospectively in this cross-sectional study between December 2009 and August 2010. Also, subjects were excluded from the study if they had conditions affecting bone metabolism. Different biochemical parameters were assayed: Testosterone, Estradiol, sex hormone binding globulin, Osteocalcin, vitamin D, crosslaps, intact parathyroid hormone and alkaline phosphatase. Dual-energy X-ray absorptiometry was used to measure the Bone mineral density (BMD) (g/cm^2^).

**Results:**

In this study, among the 142 Moroccan men, 29 (20.1%) had densitometry osteoporosis and the prevalence of vitamin D insufficiency was 94%. No correlation was found between Estradiol, Testosterone and bone mineral density but we found significant differences in the levels of Estradiol between patients with osteoporosis, osteopenia and normal patients. Bone mineral density at the lumbar spine was negatively correlated to hormone-binding globulin and positively correlated to free androgen index, free estrogen index and the Body mass index. BMD at the total hip was positively correlated to free androgen index, Body mass index and negatively correlated to sex hormone binding globulin, alkaline phosphatase, intact parathyroid hormone, osteocalcin, Crosslaps and age.

**Conclusion:**

Our study showed that increasing age, intact parathyroid hormone and alkaline phosphatase levels and decreasing body mass index were the most important independent factors associated to the presence of a low BMD at the total hip. Increasing body mass index and free androgen index level were the most important independent factors associated to the presence of a low BMD at the lumbar spine. The combination of variable that best predicted the male osteoporosis is age, body mass index, alkaline phosphatase and cigarette smoking.

## Introduction

Osteoporosis is a metabolic bone disorder characterized by low bone mass and micro- architectural deterioration, with a subsequent increase in bone fragility and susceptibility to fracture [1]. It affects 13% of Caucasian men older than 50 years (versus 40% in women) and a 15% lifetime risk of osteoporotic fractures in the same age group [2]. Gonadal steroid hormones play a crucial role during skeletal growth and maturation in both men and women [3]. In fact, hypogonadism is a well recognized risk factor for osteoporosis in aging men, and it is found in 10% to 15% of cases [4, 5]. Also, most cohort studies indicate that estrogen concentrations are associated with bone mineral density (BMD) and bone turnover as well as bone loss in aging men [4, 5]. This correlation between Estradiol (E) and bone remodeling markers has been proven by treatment of elderly men with an aromatase inhibitor resulted in significant increase in bone resorption, together with decreases in bone formation markers [6, 7]. Sex hormone-binding globulin (SHBG) is a plasma glycoprotein that binds to sex steroids, thereby regulating their bioavailability [8], and the link between SHBG, BMD and bone remodeling markers should be given a careful attention. Also, vitamin D (25(OH)D3) has been measured, because 25(OH)D3 deficiency includes secondary hyperparathyroidism, accelerated bone loss, increased bone turnover, osteoporosis and fractures. The aim of this study was to evaluate the relationship between sex hormone levels, biochemical markers of bone turnover and BMD in a population of asymptomatic men.

## Methods

### Subjects

This was a cross-sectional study conducted from December 2009 to August 2010. Men were recruited prospectively through advertisements and “word of mouth”. Men who volunteered to participate in the study were enrolled after taking an informed and written consent. One hundred and forty two consecutive men who had no previous diagnosis of osteoporosis took part in the study; all subjects were screened using a detailed questionnaire, history, behaviors and physical examination. Subjects were excluded from the study if they had conditions affecting bone metabolism, such as diseases of kidney, liver, parathyroid, thyroid, diabetes mellitus, hyperprolactinemia, rheumatoid arthritis, ankylosing spondylitis, malabsorption syndromes, malignant tumors, hematologic diseases, or previous pathological fractures. Subjects were also excluded if they had been receiving corticosteroids, thyroid hormone, fluoride, bisphosphonate, calcitonin, thiazide diuretics, barbiturates, antiseizure medications, 25(OH)D3 or calcium-containing drugs. Our institutional review board approved this study. The procedures of the study were in accordance with the Declaration of Helsinki, and local ethics committee approval was obtained for the study. Each subject completed a standardized questionnaire designed to document putative risk factors of osteoporosis. Height and weight were measured in our rheumatology center before DXA measurement, in light indoor clothes without shoes. Body mass index (BMI) was calculated by dividing weight in kilograms by height in meters squared.

### BMD measurement

Bone mineral density was determined by a Lunar Prodigy Vision DXA system (Lunar Corp., Madison, WI). The DXA scans were obtained by standard procedures supplied by the manufacturer for scanning and analysis. All BMD measurements were carried out by 2 experienced technicians. Daily quality control was carried out by measurement of a lunar phantom. At the time of the study, phantom measurements showed stable results. The phantom precision expressed as the coefficient of variation percentage was 0.08%. Moreover, reproducibility has been assessed by the same 2 technicians in clinical practice and showed a smallest detectable difference of 0.04 g/cm^2^ (spine) and 0.02 (hips). Patient BMD was measured at the lumbar spine and at the femurs (i.e., femoral neck and total hip).

### Biological measurements

All subjects had fasting blood taken in the morning. The samples were frozen and stored at -20°C and subsequently thawed and analyzed in one batch. Serum Testosterone (T), Estradiol (E), Vitamin D (25(OH)D_3_) sex hormone binding globulin (SHBG), Osteocalcin (OC), and Crosslaps (β-CTX), intact parathyroid hormone (PTHi) were measured using electrochimiluminescence immunoassay (ECLIA) technique (Cobas e601, Roche Diagnostics GmBH, Mannheim,Germany). Alkaline phosphatase (ALP) was measured by (Dimension^®^ RxL Max^®^ Integrated Chemistry System of Siemens). All the laboratory tests were subject to validation using National External Quality Assurance Schemes. The free androgen index (FAI), having been shown to be more sensitive for identifying subjects with abnormal androgen status than total or free testosterone. Free androgen index (FAI= Total testosterone/SHBG x 100) was calculated from the ratio of serum T to SHBG to give an estimate of the free circulating concentration. Similarly, the free estradiol index (FEI= Total E/SHBG * 100) was calculated from the ratio of serum E to SHBG.

### Statistical analysis

Results are presented as means (SD) and categorical variables are expressed as frequencies. To compare normal patients, patients with osteopenia and osteoporosis, chi-square test and ANOVA were used firstly. Correlations between continuous variables were calculated using Pearson correlation coefficients. Potential risk factors were entered to a stepwise conditional binary regression analysis and the resulted odds ratios with 95% confidence intervals were reported. Logistic-regression models were used to analyze the most important factors related to the presence of osteoporosis. The level for significance was taken as p ≤0.05. Excel 2007 and SPSS 15.0 were used for statistical analysis.

## Results

In this cohort of 142 men, the mean ± SD (range) age and Body mass index were 63.31±8.7 years and 26.42±4.20 kg/m^2^, respectively. Based on the diagnostic criteria of osteoporosis (T-score below -2.5 at the lumbar spine, the femoral neck or the total hip site) 44 subjects were diagnosed as normal (30.99%), 69 as osteopenia (48.59%) and 29 as osteoporosis (20.42%). In all patients, in which we found low levels of 25(OH)D_3_ ( the mean ± SD (range)) were 20.47±6.15 ng/mL. In our study, the prevalence of smoking was 52.11% and cigarette smoking was associated with a high prevalence of osteoporosis (p=0.004), and the smokers had a high level of ALP (p=0.029) and a low total hip BMD (TH_BMD) comparing to the nonsmokers (p=0.029) group. The concentrations of the biochemical parameters were compared among the three groups (normal bone mass group, osteopenia group and osteoporosis group). The comparison result showed that the plasma levels of ALP, SHBG, and OC were significantly higher in the osteoporosis group than in the other groups, but the levels of the FAI were significantly lower in the osteoporosis group compared to the other groups (Table 1).

**Table 1:** Comparison between normal patients, patients with osteopenia and osteoporosis (n=142)

	Normal (n= 44)	Osteopenia (n=69)	Osteoporosis (n=29)	p	p_1_	p_2_	p_3_
**Age (years): m (SD)**	60.87 (8.487)	63.42 (8.38)	66.93 (8.71)	0.012	NS	‘0.009	NS
**BMI (kg/m^2^): m (SD)**	28.20 (3.93)	26.08 (4.26)	24.41 (3.87)	<0.001	NS	<0.001	0.021
**PTHi ( pg/ml): m (SD)**	65.48 (30.88)	59.11 (21.73)	82.57 (53.19)	0.007	0.005	NS	NS
**ALP (U/L): m (SD)**	82.67 (21.13)	88.62 (21.15)	107.21 (32.34)	<0.001	0.002	<0.001	0.573
**SHBG (nmol/L): m (SD)**	48.77(19.93)	58.73 (25.23)	68.23 (34.75)	0.007	NS	0.006	NS
**T (ng/dL): m (SD)**	4.16 (1.64)	4.79 (1.81)	4.54 (2.24)	NS	NS	NS	NS
**FAI (ng/dL): m (SD)**	9.13 (3.24)	8.52 (3.2)	7.33 (3.83)	NS	NS	0.049	NS
**E (pg/mL): m (SD)**	22.83 (9.17)	21.33 (11.08)	27.18 (11.86)	NS	0.43	NS	NS
**FEI (pg/mL): m (SD)**	52.21 (25.64)	41.5 (25.9)	48.99 (27.37)	NS	NS	NS	NS
**β-CTx (ng/mL): m (SD)**	0.379 (0.22)	0.47 (0.31)	0.67 (0.57)	0.003	0.048	0.002	NS
**Osteocalcin (ng/mL): m (SD)**	20.01 (10.23)	24.15 (22.53)	41 (55.67)	0.011	0.036	0.011	NS
**25(OH)D3 (ng/mL): m (SD)**	18.59 (5.23)	21.4 (6.30)	21.22 (6.63)	0.041	NS	NS	0.048
**Lumbar spine BMD (g/cm^2^)**	1.232 (0.187)	0.989 (0.115)	0.844 (0.12)	<0.001	<0.001	<0.001	<0.001
**Total hip BMD (g/cm^2^): m (SD)**	1.07 (0.12)	0.89 (0.098)	0.699 (0.1)	<0.001	<0.001	<0.001	<0.001

Mean ± SD : m (SD) *: <0.05 **: < 0.001

One way analysis of variance + Tests Post hoc (Bonferroni)

**p: osteoporosis-osteopenia-normal p_1:_ osteoporosis – osteopenia p_2_ : osteoporosis - normal p_3_ osteopenia – normal**

**LS_BMD** lumbar spine bone mineral density **TH_BMD** Total hip bone mineral density **FAI** Free androgen index **SHBG** Sex Hormones Binding Globulin

**FEI** Free estrogen index **ALP** Alkaline phosphatase **PTH_i_** Parathormone (PTH) intact **OC** Osteocalcin

**β-CTX** Crosslaps **25(OH)D3** 25-hydroxyvitamin D **BMI** Body mass index **E** Estradiol **T** Testosterone

### Results of correlation analysis between bone mineral density with the biochemical parameters, BMI and age (Table 2)

Pearson correlation analysis showed significant negative correlations between the total hip BMD and the following variables ALP, PTHi, OC, β-CTX, SHBG, FAI, Age and the BMI. And positive correlations with the following variables BMI, FAI. Pearson correlation analysis showed significant positive correlations between Lumbar spine BMD and the following parameters the FAI and the BMI. And a negative correlation was found with the SHBG.

**Table 2:** Correlation between biochemical values and age, BMI and BMD (n=142)

	ALP	PTHi	OC	β-CTX	E	T	SHBG	FEI	FAI	25(OH)D_3_	Age	BMI
**PTHi**	0.237+											
**OC**	0.252+	0.666++										
**β-CTX**	0.310++	0.565++	0.879++									
**E**	0.110	0.283+	0.257+	0.234+								
**T**	0.02	0.06	0.13	0.06	0.45++							
**SHBG**	0.09	0.15	0.10	0.12	0.224+	0.562++						
**FEI**	-0.04	0.07	0.07	0.02	0.557++	-0.13	- 0.53++					
**FAI**	-0.10	-0.13	-0.003	-0.16	0.16	0.251++	- 0.483++	0.567++				
**25(OH)D_3_**	-0.04	-0.07	0.10	0.09	0.192+	0.190+	0.07	0.08	0.12			
**Age**	0.10	0.13	-0.04	0.06	0.10	-0.03	0.330++	-0.214++	-0.485++	-0.13		
**BMI**	-0.09	0.01	-0.173+	-0.211+	-0.12	-0.339++	-0.253++	0.13	0.05	-0.177+	-0.09	
**LS_BMD**	-0.12	0.02	-0.06	-0.13	0.05	-0.03	-0.220++	0.15	0.189+	-0.12	-0.14	0.369++
**TH_BMD**	-0.353+	-0.311++	-0.300++	-0.338++	-0.15	-0.15	-0.262++	0.09	0.169+	-0.06	-0.261++	0.369++

+ : <0.05

++ : < 0.001

### Results of correlation analysis between sexual hormones with blood biochemical parameters, BMD, BMI and age (Table 2)

Pearson correlation analysis showed that there were positive correlations between estradiol and the following variables β-CTX, OC, PTHi, and there were negative correlations between estradiol and the following variables T, SHBG and vitamine D. There was a significant significant positive correlation between the T and the BMI. and a significant negative correlation was found with the BMI. There were significant negative correlations between the FAI and the age, and between the FEI and the age too.

### Results of correlation analysis between vitamin D with blood biochemical parameters, BMD, BMI and age (Table 2)

There was a significant positive correlation between the 25(OH)D_3_ and the following parameters E, T. A significant negative correlation was found with the lumbar spine BMD.

### Multiple regression analysis with bone mineral density as dependent variable (Table 3)

Multiple regression analysis was therefore performed to determine the combination of variables that accounted for the greatest proportion of variance in BMD at each site. PTHi, alkaline phosphatase, age and Body mass index were related to total hip BMD. BMI and free androgen index were significantly related to Lumbar spine BMD.

**Table 3:** Multiple linear regression analysis with BMD as dependent variable (n=142)

TH_ BMD	Bêta	SE	p-value
BMI	0.334	0.003	<0.001
ALP	-0.218	<0.001	0.001
PTHi	-0.231	<0.001	0.002
AGE	-0.174	0.001	0.015
LS _BMD	Bêta	SE	p-value
BMI	0.360	0.004	〉0.001
FAI	0.171	0.005	0.020

### Multiple logistic regressions

To determine the combination of variables that best predicted the male osteoporosis, a multiple logistic regression analysis was performed (Table 4) and the best model comprised ALP, Age, BMI and cigarette smoking.

**Table 4:** Multiple logistic regression analysis with the presence of osteoporosis as dependent variable (n=142)

	Exp(B)	[95% Conf. Interval]	p-value
ALP	1.0255	[1.0062-1.0453]	0.0096
Age	1.0807	[1.0172-1.1481]	0.0120
BMI	0.8407	[0.7293-0.9691]	0.0167
Cigarette smoking	1.8557	[1.0488-3.2833]	0.0337

## Discussion

We analyzed in this study the association between sex hormone levels, biochemical markers of bone turnover and BMD in healthy Moroccan men. We found a higher prevalence of osteoporosis (20.45%) than what was reported in the normal Moroccan population [9], and among Caucasian men older than 50 years (13%) [2], which was probably due to a hazard effect and to the small number of men enrolled in this study. Our observations in terms of BMI are very similar to those of most studies showing that lower BMI scores were associated with BMD loss [9, 10]. The BMI of the osteoporosis group was slightly lower compared with those of the osteopenia and normal groups, and these differences were significant [11]. There were negative correlations between the BMI and OC, BMI and β-CTX, BMI and T, BMI and 25(OH)D_3_. Thus, high BMI was associated with low levels of bone remodeling markers, Testosterone and 25(OH)D_3_. Overweight may protect men against bone loss, several explanations have been proposed. First of them by increasing the amount of biologically available estrogens, in fact, estrogen is known to inhibit bone resorption by osteoclasts [12]. A larger body mass imposes a greater mechanical loading on bone and that bone mass increases to accommodate the greater load. Although smoking is often cited as a risk factor for osteoporosis, the influence of smoking on osteoporosis remains unclear. In our study the prevalence of smoking was 52.11% and cigarette smoking is associated with a high prevalence of osteoporosis (p=0.004), and smokers had a high level of ALP (p=0.029) and a low TH_BMD comparing to nonsmokers (p=0.029). Pearson correlation analysis in the group of smokers showed that there were positive correlations between the contents of ALP and these parameters β-CTX, OC and PTHi. Thus, smoking was associated with high level of bone turnover markers.

Smoking is thought to cause low bone density through a combination of different mechanisms, smoking has been linked to a decrease in parathyroid hormone and estrogen levels as well as to an increase in the level of cortisol and adrenal androgens, changes that have been linked to an increased risk of osteoporosis [13]. Smoking reduces body mass, which is postulated to provide an osteogenic stimulus and is linked to higher BMD [14]. Smoking reduces the level of 25(OH)D3 in the body [15]. Smoking increases free radicals and oxidative stress which affects bone resorption [16]. Smokers are more likely to suffer from peripheral vascular disease, reducing blood supply to the bones [17]. Finally, there may also exist direct toxic effects of many of the constituents in tobacco smoke on bone cells [18]. Smoking is significantly associated with higher level of alkaline phosphatase in men [19]. Thus, In our study smokers had a high level of ALP (p=0.029). We do not find any significant difference in E, T levels between patients in osteoporotic group, osteopenic group and normal group nor any correlation between blood E and BMD whatever the site measured. Slemenda and al found that BMD measurements at the hip and spine correlated negatively with serum T levels (correlation coefficients varying from -0.20 to -0.28, p= 0.03-0.10) [20]. “Legrand and al” and “Lomereau and al” did not show any statistical significant difference in E levels between controls and osteoporotic patients, and the blood E did not correlate with spine BMD [11, 21]. In contrast, several cross-sectional studies have shown a significant correlation between E levels and bone mass in men [5, 22–24]. FAI correlated positively to BMD but greater at the lumbar spine. FEI correlated weakly to the BMD at the lumbar spine. “Gennari and al” found that the free estradiol index (FEI) correlates positively with BMD values at the femur and lumbar spine [24]. Pearson correlation analysis showed that there were positive correlations between the contents of E and these parameters PTH, OC and β-CTX, but negative correlations between the FAI and age, FEI and age, BMI and OC, BMI and β-CTX, BMI and T. The association between E and bone resorption markers has been confirmed in another study where significant correlations were found only with markers of bone resorption (serum and urinary NTX), but not with biochemical indices of bone formation (OC), bone alkaline phosphatase isoenzyme (BAP) [25]. Moreover, prospective studies showed that serum E was a better predictor than serum T of both the increase in bone loss in elderly men [24, 26]. In a cross-sectional study that included men and women, “Khosla and al” reported inverse correlations between urinary NTX (cross-linked N-terminal telopeptides of type I collagen) levels and both “bioavailable” E and “bioavailable” T [12]. “Szulc and al”found that only bioavailable E levels in men were negatively correlated with bone turnover, but no associations were observed with total E, or any testosterone measure [27].

The impact of serum testosterone on bone health parameters appears less significant and uncertain [28]. The current evidence suggests that E plays a greater role in maintenance of skeletal health than testosterone, but that androgens also have direct beneficial effects on bone. Testosterone is metabolized via the cytochrome P450 aromatase enzyme complex into 17β-estradiol, and increasing evidence indicates that at least part of the effect of androgens on bone is mediated by their aromatization to estrogens [29, 30]. And the other effects of the androgens by androgen receptors are present on bone cells, and androgen receptor mediated actions on bone have been known for several years [31]. In our data SHBG was positively correlated to age and negatively correlated to BMI and BMD (lumbar spine and total hip). Thus, the concentration of SHBG rises with age and the levels of SHBG are low in obese patients and extremely high in patients with anorexia nervosa [31]. Our study and several case-control studies have demonstrated no difference in total sex steroids, but significantly higher SHBG and lower FAI in osteoporotic group [30–32]. “Legrand and al” observed higher levels of plasma SHBG in two thirds of the 80 osteoporotic patients in their study. SHBG correlated negatively with BMD at the femoral neck, both in idiopathic osteoporosis (r=-0.34, p <0.01) and secondary osteoporosis (r=-0.34, p < 0.01) [21]. Likewise, “Evans and al” found a significant increase in SHBG levels in 81 men with both idiopathic osteoporosis and fractures as compared with 68 healthy controls [31]. Longitudinal study shows that SHBG correlated negatively with trabecular bone mass [32].

We defined the hypovitaminosis D as a circulating level of 25(OH)D3 below 30 ng/mL. In this study, the prevalence of hypovitaminosis D was 94%, similar to results of previous studies of vitamin D prevalence in Moroccan women. Although Morocco enjoys a sunny climate throughout the year, many factors may explain these high prevalences. It may be related to low vitamin D intake and in our country, food is not supplemented with vitamin D. Hence, cutaneous synthesis would be the major source of 25(OH)D3. This high prevalence of hypovitaminosis D was also reported in a cross-sectional study among Saudi Arabian men (87.8%) [33]. Also, we got significant positive correlation either between 25(OH)D3 levels and E,T and the BMI. 25(OH)D_3_ was negatively correlated to age, hence, men with low 25(OH)D_3_ were older and with low level of T and E. A recent randomized placebo-controlled trial by “Pilz and al” suggested that 25(OH)D_3_ may increase the production of T in men [34]. “David M Lee and al” in a cross-sectional study of 3369 community-dwelling men aged 40-79 years in eight European centers, 25(OH)D3 was positively associated with total and free T and negatively with E and LH in age- and centre-adjusted linear regressions, this result was in contrast to our finding for E [35]. We did not find any correlation between 25(OH)D_3_ and BMD, some studies found a positive relationship between serum 25(OH)D3 and BMD, but others did not find any relationship neither [36, 37]. Perhaps because of differing age, race, gender and health/nutritional status in this reported cohorts. In our data not all patients with vitamin D insufficiency (25(OH)D_3_ < 30 ng/l) develop secondary hyperparathyroidism but only 50%. In fact, vitamin D insufficiency and PTH over the 65 ng/l, this may be a threshold for initiating the optimal replacement therapy [38]. The TH_BMD correlate negatively with SHBG, OC, PTH, β-CTX, ALP, age and positively with FAI, BMI. The LS_BMD was negatively correlated to SHBG and positively correlated to FAI, FEI and the BMI. BMI was the best predictor of BMD at all sites. In fact, the relationship between markers of bone remodeling and BMD in men varies in the literature, it is usually weak [35, 36] but appears stronger in subjects over 50 years of age. Some authors do not find any significant relationship between such markers and BMD in men, whatever their age [39].

Our study demonstrated that increasing age, PTHi and ALP levels and decreasing BMI were the most important independent factors associated with the presence of a low bone mineral density at the total hip. Increasing BMI and FAI level were the most important independent factors associated with the presence of a low bone mineral density at the lumbar spine. The best model that can predict osteoporosis in Moroccan men was the combination of the ALP, Age, BMI and cigarette smoking. Our study has strengths and limitations. All of the DXA and biochemical measurements were conducted with a single bone densitometer and a single biochemistry laboratory, with very careful quality controls in place. The main limitations lie in the cross-sectional nature of the study and in the procedures used to select subjects, who were all volunteers and ambulatory. The study population had a higher prevalence of osteoporosis which was probably due to a hazard effect.

## Conclusion

BMI was the best predictor of BMD at all sites. Age, PTHi, ALP and BMI were the best predictors at the TH_BMD and BMI, FAI were related to LS_BMD. The combination of variable that best predicted the male osteoporosis is Age, ALP, BMI and cigarette smoking. 94% of the patients in this study had a vitamin D insufficiency. Furthermore, a positive correlation was found between 25(OH)D_3_ and E,T and negative correlation was found with the age. However, large scale longitudinal studies are needed to further evaluate the relationship between this parameters and the BMD.
